# Localized oncolytic virotherapy inflames distant tumors and synergizes with immune checkpoint blockade leading to systemic tumor rejection

**DOI:** 10.1186/2051-1426-1-S1-O9

**Published:** 2013-11-07

**Authors:** Dmitriy Zamarin, Rikke Holmgaard, Sumit Subudhi, Mena Mansour, Peter Palese, Taha Merghoub, Jedd D Wolchok, James P Allison

**Affiliations:** 1 Medicine, Memorial Sloan-Kettering Cancer Center, New York, NY, USA; 2 Immunology, MD Anderson Cancer Center, Houston, TX, USA; 3 Microbiology, Mount Sinai School of Medicine, New York, NY, USA

## 

Preexistent tumor inflammation is associated with superior clinical benefit from immunotherapies targeting immune checkpoints, calling for development of strategies that can induce tumor immune infiltration. To this end, therapy with oncolytic viruses (OV) presents an attractive strategy, though clinical development of OV’s has been largely hampered by their poor systemic delivery to metastatic sites. Newcastle Disease Virus (NDV) is a member of the Avulavirus genus in the Paramyxoviridae family, which has been shown to infect a number of avian species, but to cause no disease in humans. For the past 50 years, NDV has been shown to be an effective oncolytic agent, causing specific lysis of cancerous but not normal cells. During an effort to develop a systemically-active oncolytic NDV, we found that localized therapy with the virus induced systemic tumor inflammatory response with activated effector but not regulatory T cell infiltration in distant tumors without virus spread to these sites. Combination therapy with localized NDV and systemic CTLA-4 or PD-1 blockade led to antigen-dependent distant tumor rejection in both NDV-susceptible (B16) and resistant (TRAMP C2 and CT26) tumor models, and protected the animals from further tumor re-challenge. This effect was associated with marked distant tumor infiltration with activated effector, but not regulatory T cells, and was dependent on CD8 and NK cells, and types I and II interferons. To further enhance the T cell effector function in the treated tumors, we genetically engineered a panel of oncolytic NDV’s expressing co-stimulatory ligands. We specifically focused on the inducible co-stimulator (ICOS), which in clinical trials has been associated with improved clinical benefit from anti-CTLA-4 therapy. When used in combination with anti-CTLA-4 therapy, NDV expressing ICOS ligand (NDV-ICOSL) induced rejection of distant tumors more efficiently than the parental virus, leading to long-term animal survival in the majority of animals and protection against tumor re-challenge. Our findings uncover the mechanisms of NDV-induced systemic anti-tumor immune responses and provide a strong rationale for clinical exploration of combination therapies of engineered oncolytic viruses with agents targeting immune checkpoints.

